# Effects of Dimethyl Sulfoxide in Cholesterol-Containing Lipid Membranes: A Comparative Study of Experiments *In Silico* and with Cells

**DOI:** 10.1371/journal.pone.0041733

**Published:** 2012-07-25

**Authors:** Marie-Amélie de Ménorval, Lluis M. Mir, M. Laura Fernández, Ramon Reigada

**Affiliations:** 1 Université Paris-Sud, Laboratoire de Vectorologie et Thérapeutiques Anticancéreuses, UMR 8203, Orsay, France; 2 CNRS, Orsay, Laboratoire de Vectorologie et Thérapeutiques Anticancéreuses, UMR 8203, Orsay, France; 3 Institut Gustave Roussy, Laboratoire de Vectorologie et Thérapeutiques Anticancéreuses, UMR 8203, Villejuif, France; 4 Laboratorio de Sistemas Complejos, Departamento de Computación, Facultad de Ciencias Exactas y Naturales, Universidad de Buenos Aires, Buenos Aires, Argentina; 5 Consejo Nacional de Investigaciones Científicas y Técnicas, Buenos Aires, Argentina; 6 Department de Química Física and Institut de Química Teòrica i Computacional, Universitat de Barcelona, Barcelona, Spain; Consiglio Nazionale delle Ricerche, Italy

## Abstract

Dimethyl sulfoxide (DMSO) has been known to enhance cell membrane permeability of drugs or DNA. Molecular dynamics (MD) simulations with single-component lipid bilayers predicted the existence of three regimes of action of DMSO: membrane loosening, pore formation and bilayer collapse. We show here that these modes of action are also reproduced in the presence of cholesterol in the bilayer, and we provide a description at the atomic detail of the DMSO-mediated process of pore formation in cholesterol-containing lipid membranes. We also successfully explore the applicability of DMSO to promote plasma membrane permeability to water, calcium ions (Ca^2+^) and Yo-Pro-1 iodide (Yo-Pro-1) in living cell membranes. The experimental results on cells in culture can be easily explained according to the three expected regimes: in the presence of low doses of DMSO, the membrane of the cells exhibits undulations but no permeability increase can be detected, while at intermediate DMSO concentrations cells are permeabilized to water and calcium but not to larger molecules as Yo-Pro-1. These two behaviors can be associated to the MD-predicted consequences of the effects of the DMSO at low and intermediate DMSO concentrations. At larger DMSO concentrations, permeabilization is larger, as even Yo-Pro-1 can enter the cells as predicted by the DMSO-induced membrane-destructuring effects described in the MD simulations.

## Introduction

Dimethyl sulfoxide (DMSO, (CH_3_)_2_SO) is a small amphiphilic molecule that is traditionally used as a cryoprotectant [1], solvent for peptides in NMR studies [2], cell fusogen [3], and chemical penetration enhancer to deliver active molecules through the skin and into the cells [4]. Experimental studies devoted to the investigation of the specific changes of lipid in membranes in the presence of DMSO have revealed that this compound replaces water in the inner region of the lipid headgroup [5] and causes an increase of area per lipid and a decrease of membrane thickness [6]. These effects have been confirmed by means of Molecular dynamics (MD) simulations in a series of recent articles. Sum and de Pablo demonstrated that DMSO is preferentially placed below the headgroup of the membrane lipids [7]. A similar behavior is reported in simulations of ceramide bilayers [8], where DMSO is shown to promote phase transition from gel to liquid crystalline phase. All these observations suggest an increase of the membrane permeability in the presence of DMSO that has been already confirmed by simulations. Anwar et al. showed a DMSO-mediated enhancement of the permeability through the formation of water pores, both in atomistic [9], [10] and coarse-grained [11] MD simulations.

Although the MD studies mentioned above have already addressed this issue, there is an important actor that has been missing in all these studies: cholesterol (Chol). Chol is the most common lipid component in animal cell membranes and its content ranges depend on the cell type. For example, in CHO cells (Chinese Hamster Ovary cells, same species than the cells used in our experiments), Chol can represent up to a 30 mol% of the total membrane lipid molecules [12]. Chol is fundamental for determining many structural properties of the cell membrane: by means of its condensing effect, inclusion of Chol results in more densely packed and ordered membranes, thus dramatically reducing their spontaneous permeation to small molecules. Due to these strong effects on membrane cohesion, it is not obvious that the mechanisms of action of DMSO captured so far by MD simulations in single-component bilayers are also instrumental in Chol-containing bilayers. The understanding of the molecular details of the action of DMSO in living mammalian cell membranes requires, therefore, the study of Chol-containing lipid bilayers.

In this context, the ability of DMSO to facilitate transport across the cell membrane has been rather unexplored so far. Actually, despite the huge potentiality of the membrane permeabilizing effect of the DMSO, only sporadic and specialized experimental works have been devoted to it without investigations about the mechanism involved [13]. Our first motivation is then to propose that DMSO could be exploited in processes that involve transport across the cell membrane in order to improve or even develop new drug delivery approaches or new transfection techniques in laboratory and clinical treatments. Here, we contribute to this issue with complementary approaches that cover a broad perspective ranging from molecular simulations at the atomic level to the experimental implementation in living cells.

In this paper we report the main observations from MD simulations of Chol-containing dioleoylphosphatidylcholine (DOPC) bilayers under the influence of DMSO. The main qualitative behaviors reported previously [7]–[11] for single-lipid bilayers are captured here for Chol-containing membranes and in particular we characterize the three regimes of action of DMSO as a function of its concentration in the solvent phase [9]–[11]. Interestingly, pore formation in DOPC/Chol bilayers is reported at intermediate DMSO fractions, and a close inspection of simulation trajectories allows the description of a molecular-detailed mechanism that discloses the role of each of the system components in the pore formation process. Particularly, DMSO provides a new poration mechanism that facilitates water to penetrate the hydrophobic region of the membrane, thus increasing the occurrence of pore formation.

Experiments with living cells were conducted to reproduce the main observations of numerical simulations. It is the first time that experiments trying to analyze the DMSO permeability are systematically conducted. We decided to study the effects of different DMSO concentrations on the cell membrane permeability of three markers of different sizes. Different experiments using a fibroblast cell line are reported here, displaying permeabilization to water, Ca^2+^ ions and Yo-Pro-1 for a range of DMSO concentrations. These experiments not only demonstrate the permeabilization due to DMSO in living cells, but they also reveal, as a function of the DMSO concentration, the different behaviors that were predicted from the *in silico* (numerical) observations.

## Results and Discussion

### Molecular Dynamics Simulations

#### Three modes of DMSO action

**Figure 1:**
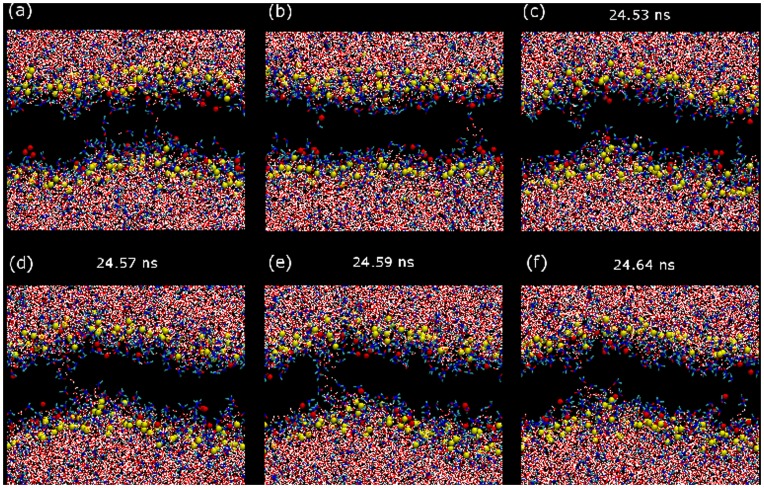
Fluctuations and transient water pores. Snapshots in the (x,z) view for the dynamics of a DOPC/20%Chol bilayer with 10 mol% DMSO. The first two snapshots illustrate the existence of water fluctuations that promote the presence of single (a) or groups (b) of a few water molecules in the hydrophobic region of the membrane. (c–f) Sequence of the formation and collapse of a transient water pore. Water molecules are plotted with red and white sticks whereas red, blue and green sticks are used for DMSO molecules. DOPC and Chol molecules are not plotted except for their phosphate (yellow beads) and hydroxyl (red beads) groups, respectively.

MD Simulations are run for DOPC membrane systems mixed with 20 mol% of Chol and different molar fractions of DMSO in the solvent mixture. As reported in Ref. [9], three distinct modes of action are observed depending on DMSO concentration. At low DMSO fractions (≤10 mol%, regime I), membranes experience significant structural changes (see below). In this regime, only sporadic and very transient hydrophobic pores can be arranged in form of single-molecule water columns that cross the membrane and rapidly disappear (Figure 1). We have observed that the occurrence of transient pore formation increases with DMSO content.

**Figure 2:**
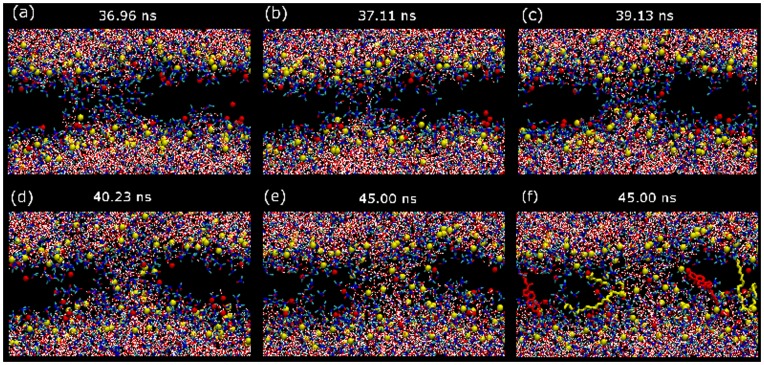
Sequence of stable pore formation. (a–e) Snapshots in the (x,z) view for the dynamics of formation of a stable hydrophilic water pore in a DOPC/20%Chol bilayer with 15 mol% DMSO. Water molecules are plotted with red and white sticks whereas red, blue and green sticks are used for DMSO molecules. DOPC and Chol molecules are not plotted except for their phosphate (yellow beads) and hydroxyl (red beads) groups, respectively. (f) The same as in panel (e) but some representative DOPC (yellow sticks) and Chol (red sticks) molecules forming (the ones parallel to the bilayer plane) and non forming (the ones perpendicular to the bilayer plane) the hydrophilic pore are plotted.

At intermediate DMSO concentrations (regime II), membranes display the previous behavior during a few nanoseconds until stable pores are formed. The sequence of pore formation is well known [9]: an initial water fluctuation crosses the whole membrane and a hydrophobic pore is formed, the lipid molecules around it rotate and cover the water filament with their hydrophilic headgroups forming a hydrophilic pore that rapidly widens and becomes stabilized (see Figure 2).

At large DMSO amounts (≥40 mol%, regime III), the lipid bilayer first displays the effects reported in regime I, until extreme undulations and multiple pore formation destroy the membrane configuration (not shown).

#### Low DMSO fractions already affect the main structural membrane properties

At low DMSO fractions (≤10 mol%), the simulated membranes display structural modifications but still preserve their bilayer conformation (no pore is formed yet). A first inspection of the simulated membranes shows that they become more undulated after addition of DMSO. Although this flexibility gain has not been particularly quantified, it can be related to a series of molecular/microscopic membrane properties that are summarized in this section.

**Figure 3:**
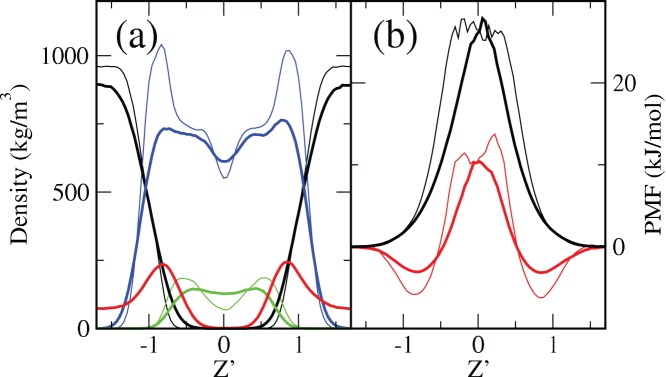
Mass density and membrane permeability profiles. (a) Mass density and (b) potential of mean force (PMF) profiles in a DOPC/20%Chol bilayer. Thick lines correspond to 5 mol% DMSO systems, whereas thin curves stand for 0 and 1 mol% DMSO in the (a) and (b) panels, respectively. The profiles correspond to water (black), DOPC (blue), Chol (green) and DMSO (red) and are represented in a scaled distance respect to the bilayer center where the maxima of phosphate groups are fixed at Z’ = ±1.

As anticipated above, even if initially placed in the bulk aqueous phase, DMSO molecules partially penetrate the membrane. The analysis of the mass density profiles plotted in Figure 3a reveals that DMSO is preferentially located in the inner membrane/water interface region, close to the phosphate groups as it corresponds to its amphiphilic nature. Figure 3a also depicts that the incorporation of DMSO to the membrane breaks its transversal ordering: in the absence of DMSO lipid profiles show the typical features obtained from diffraction experiments (two pronounced lipid density peaks in the phosphate group positions and a minimum in the middle of the bilayer), whereas upon addition of DMSO, lipid density profiles are smoothed. It has to be also noticed that the addition of DMSO increases water penetration into the membrane (Figure 3a), so it seems that DMSO replaces water in the interfacial regions but does not totally exclude it.

The equilibrium values for the main structural properties of the simulated membranes are provided in Table 1. As anticipated, addition of DMSO increases the total simulated membrane area (see area in Table 1) and reduces the bilayer thickness, here estimated as the average distance between phosphorous atoms in opposite leaflets (see P-P distance in Table 1). For example, addition of 5 mol% of DMSO in the solvent phase causes an increase of about 30% in membrane area and a reduction of 13% in thickness.

**Table 1:** Averaged properties characterizing the simulated DOPC/20%Chol bilayer systems.

%mol DMSO	Area (nm^2^)	P-P dist (nm)	<-S_CD_>	Lipid tail angle (°)	Chol angle (°)
0	44.5	4.17	0.1528	28.26	30.88
1	47.5	3.93	0.1503	30.62	29.27
2.5	52.5	3.70	0.1205	34.76	31.89
5	58.1	3.60	0.0994	38.17	31.05
7.5	63.9	3.25	0.0809	41.09	34.84
10	68.4	3.14	0.0775	41.65	39.20

The internal ordering of lipid membranes is typically quantified by means of the deuterium order parameter, -S_CD_ [14]. Larger values for -S_CD_ imply higher chain ordering. The average order parameter <-S_CD_>, taken as the average for all CD segments, quantifies globally the disordering effect. Addition of 5 mol% of DMSO decreases chain ordering by 40% in 20 mol% Chol bilayers (see Table 1). A closer inspection of S_CD_ profiles along lipid chains (not shown) reveals how DMSO has a strong disordering effect in all carbon segments, not only in those close to the inner membrane/water interface where DMSO is accumulated. Therefore, DMSO increases the membrane area and leaves more free space inside the whole hydrophobic region of the membrane, so that lipid tails become less packed and freer to adopt more disordered configurations.

The orientation of the lipid components is also a common outcome of the internal membrane order. Ordered membranes normally display lipids rather oriented in the bilayer normal direction. The orientation of an acyl chain can be quantified by its tilt angle with respect to the bilayer normal. The average values for the lipid tail tilt angle are presented in Table 1 and corroborate the above-mentioned observations: addition of DMSO inclines DOPC molecules. Chol tilt is also increased upon addition of DMSO (Table 1), reflecting that its ability to order the membrane has been reduced [15].

**Figure 4:**
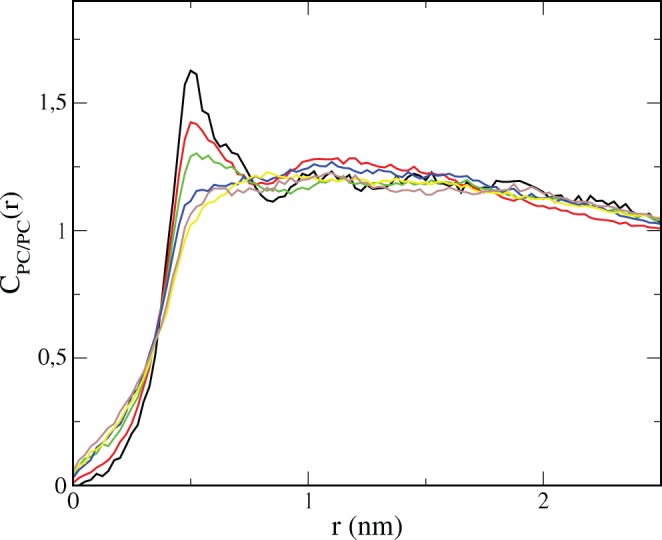
Lateral ordering of lipid tails. Spatial pair density correlation functions of the PC acyl chains, C_PC/PC_(r), for the simulated DOPC/20%Chol bilayers under the influence of different amounts of DMSO. The color code indicates the DMSO content: 0 mol% (black), 1 mol% (red), 2.5 mol% (green), 5 mol% (blue), 7.5 mol% (yellow) and 10 mol% (maroon).

Another important issue on membrane structure is its lateral organization. To quantify the lateral order, spatial pair density correlation functions C_PC/PC_(r) have been computed and plotted in Figure 4. These functions measure the density variation of the center of mass of DOPC molecules with respect to its average density as a function of the in-plane distance r from the center of mass of a DOPC molecule. Spatial ordering is observed as correlation peaks that correspond to the different coordination shells. As it is observed in Figure 4 spatial correlation is clearly lost upon addition of DMSO.

Although stable pores are not still formed at the DMSO fractions reported in this section, membrane permeability displays an increase upon addition of DMSO. Membrane permeability to a particular compound can be characterized by the variation of free energy as a function of the position of a molecule of this compound along the bilayer normal, the so called potential of mean force (PMF). This function, ΔG(z), can be computed from the mass density profiles, **ρ**(z), obtained in MD simulations using 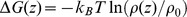 where **ρ**_0_ is the average density of the analyzed compound in the bulk phase. A large barrier of the PMF implies that the membrane is rather impermeable to the analyzed compound. The PMF profiles are plotted in Figure 3b for different amounts of DMSO and show that water and DMSO molecules can be eventually transported across the simulated DOPC/20%Chol bilayers. In the hydrophobic region of these membranes, water and DMSO display an energy maximum that is reduced after addition of DMSO (see Figure 3b). The activation energy for water is always larger than for DMSO, but becomes reduced after addition of DMSO; namely, DMSO permeabilizes lipid membranes to water. The passage of water molecules across the membrane is performed in two different ways. One way is that single water molecules cross the membrane, whereas the other mechanism implies a collective process to form a water-column pore that traverses the hydrophobic region of the membrane (transient hydrophobic pore). These transient hydrophobic pores are reminiscent of the recently described nanopores or electropores in MD simulation of membranes exposed to large voltage differences [16], [17]. Addition of DMSO molecules promotes the latter mechanism (see Figure 1). No lipid headgroup collective reorientation is observed during the formation process of this kind of transient pores. As explained above, further addition of DMSO may result in stable (hydrophilic) pore formation.

So far, all the results reported in this section evidence that the role of DMSO is to counteract the condensing effect of Chol. Whereas Chol increases membrane packing, ordering and cohesion, DMSO has the opposite effect.

#### Molecular mechanism of DMSO-mediated pore formation

**Figure 5:**
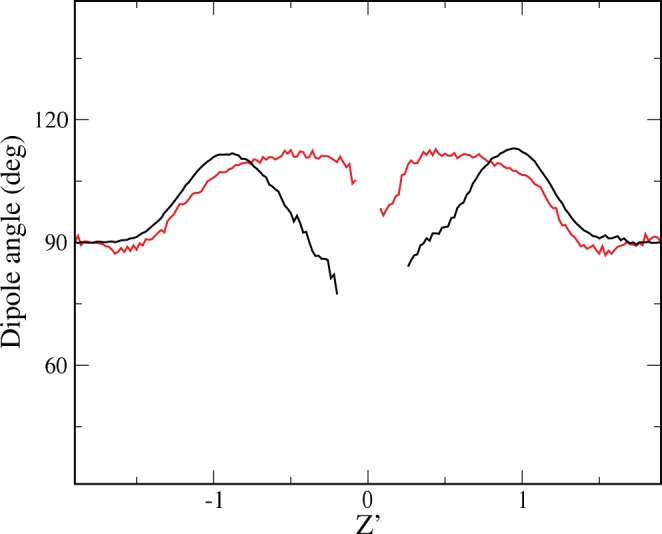
Preferential orientation of DMSO and water molecules. Membrane profiles of the dipole orientation angle respect to the bilayer normal (pointing outwards at each leaflet) of DMSO (red) and water (black) molecules. In both cases the dipole vector points to the positively charged part of the analyzed molecule. Results from a DOPC/20 mol%Chol bilayer with 5 mol% DMSO in the solvent mixture. The profiles are represented in a scaled distance respect to the bilayer center where the maxima of phosphate groups are fixed at Z’ = ±1.

The molecular characteristics of DMSO provide this compound with the particular abilities that have been reported so far in this paper, and that are fundamental to depict the molecular mechanism of DMSO-mediated pore formation. Its small size and its amphiphilic nature facilitate its interaction with the different groups coexisting in the inner membrane/water interface. In this location, it can be conjectured that the hydrophilic part of DMSO interacts with water and polar head groups, whereas the two hydrophobic methyl groups interact with the hydrophobic inner membrane region. This hypothesis is confirmed by computing the average profile orientation of the vector connecting the O and S atoms of DMSO (dipole vector pointing from the negative to the positive parts of the molecule). In Figure 5, the average angle of this vector with the bilayer normal (pointing outwards at each leaflet) is plotted for a DOPC/20%Chol membrane system with 5 mol% DMSO, although the general features are preserved for other solvent compositions. A random orientation of the dipole vector would result in an average angle of 90°. However, the average dipole angle is larger than 90° in both leaflets, and this means, as speculated above, that DMSO is preferentially aligned in such a way it approaches its O atom to the lipid polar groups and places their methyl groups in the more hydrophobic region of the interface.

Water molecules also display a preferential orientation close to the membrane interface. In Figure 5 the orientation profile for the water dipole (again defined pointing from the negative to the positive charged part of the molecule) respect to the bilayer normal is also presented. In both leaflets, water molecules located close to the membrane/water interface are aligned with their H atoms pointing to the membrane.

The preferential orientation of DMSO molecules in the membrane interface is crucial to understand the permeability gain reported so far. First, the increase of water penetration observed in mass density and permeability profiles (Figure 3) is mediated by the presence of DMSO in the interface membrane region: DMSO occupies the inner interface region, but due to its dual character it acts as a surfactant and stabilizes the presence of water molecules. Namely, DMSO partially dehydrates the lipid polar region but increases the water presence in more inner parts of the membrane interface well below the phosphate groups. Second, a detailed inspection of the transient water pores formed in regime I (see Figure 1) reveals that DMSO molecules wrap the initial water fluctuations and the ensuing transient water columns, thus promoting the permeability gain observed in Figure 3.

**Figure 6:**
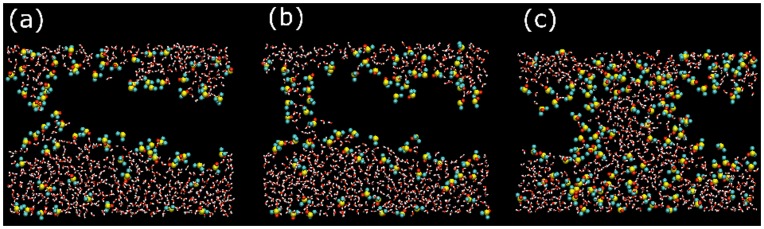
DMSO stabilizes water fluctuations and pores. Snapshots in the (x,z) view for system slices with thickness 0.4 nm in the ‘y’ coordinate. Water molecules are plotted with red and white sticks, whereas beads are used for DMSO molecules. For clarity, lipids are not plotted. (a) Detail of the initial water fluctuation in the Figure 1c. (b) Detail of the water column in Figure 1e. (c) Detail of the stable pore in Figure 1e.

Finally, the formation of stable water pores in regime II is also directly mediated by DMSO. The molecular mechanism visualized in Figure 2 shares some common features with pore formation in electroporation conditions [18], [19]. First, the disordering of the water molecules at the membrane interface may eventually generate a water defect that initiates pore formation. Then, such water defect can develop into a water column penetrating into the inner region of the bilayer (hydrophobic pore). Further on, the lipid head groups (including the hydroxyl groups of Chol molecules) reorient in order to cover the initial water column and form a hydrophilic pore (see the sequence in Figure 2). As a difference with electroporation, here DMSO plays a crucial role in the initiation, growth and stabilization of pores. In Figure 6 this role is clearly illustrated for the examples in Figures 1 and 2 by plotting cross-section snapshots of the simulated systems at different pore formation steps. In a first stage, DMSO stabilizes the initial water fluctuations with a sort of envelope that act as a surfactant protection (see Figure 6a). In a second step, the water column is wrapped in a similar manner by DMSO molecules so its hydrophobic character is tempered and becomes stable for longer time (see Figure 6b). Finally, lipid head groups rotate to form the hydrophilic pore, but even in this final situation, DMSO molecules are still located surrounding the water pores (see Figure 6c).

The observations reported in this section indicate that DMSO-induced permeability effects are not only caused by the modification of membrane properties that, in turn, facilitate the water passage across the membrane. Instead, we have demonstrated that permeability is mediated directly by DMSO. Namely, DMSO provides an exclusive mechanism of membrane permeability whose molecular details have been unveiled in this paper. This mechanism is rather independent of membrane composition: it has been presented here for DOPC/20%Chol bilayers, but it is also reproduced in DOPC/40%Chol and Chol-free systems (not shown).

### Experiments with Living Cells

To confirm the results obtained by the MD simulations, experiments with living cells were performed. Three markers of permeabilization were studied: water, Ca^2+^ and Yo-Pro-1. The crossing of water through the cell plasma membrane was first assessed as it is a common phenomenon studied in MD simulations [20]–[22]. The observations made on water (MW = 18 g/mol) were then corroborated by monitoring the Ca^2+^ (MW = 40.1 g/mol) entry in the cell. Finally, the extent of the permeabilization was estimated by assessing the entrance of Yo-Pro-1 (Yo-Pro-1 iodide MW = 629 g/mol), a non-cell permeant molecule also used as a permeabilization marker [23], [24]. This is a larger marker than the calcium ion.

#### Permeabilization to water at different concentrations of DMSO

**Figure 7:**
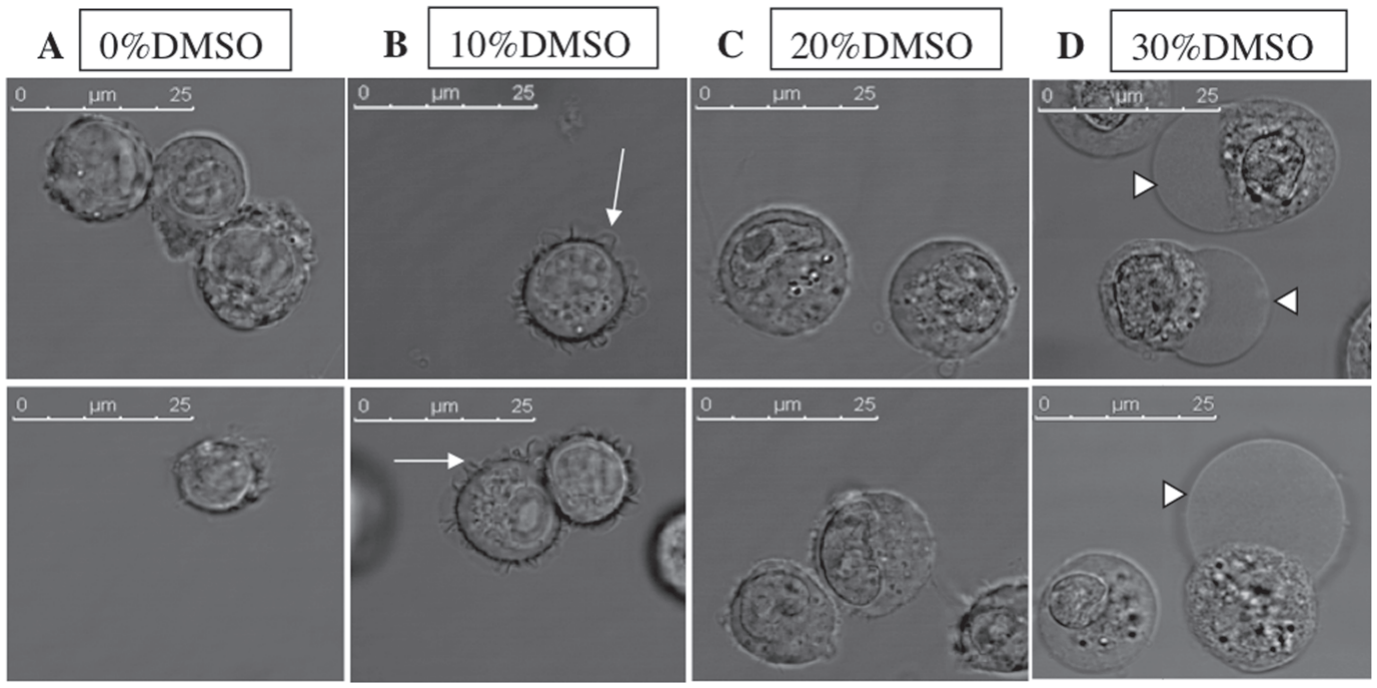
Morphology of DC-3F cells in bright field microscopy. For A, B, C and D the upper and the lower panels are two different fields on the same slide. (A) DC-3F cells without DMSO. (B) Cells in the presence of 10% of DMSO with membrane undulations (arrows). (C) Round and swollen cells in the presence of 20% of DMSO. (D) Cells in the presence of 30% of DMSO presenting blebs (arrows heads). Cells were observed with a 63x objective. DMSO was diluted in complete MEM. Pictures were taken after 1 h of incubation in the different solutions.

Cell permeabilization to water was monitored by microscopy. If the cells are permeabilized to water, they will shrink or swell, depending on the interior and exterior osmotic pressures, which will affect the global shape of the cells and their volume. The aspect of the cells after one hour of incubation in the presence of different concentrations of DMSO is shown on Figure 7. After one hour in the presence of 10 vol% of DMSO (2.74 mol%), small undulations of the plasma membranes and the absence of swelling indicated that the membrane is affected but there is no massive entry of water molecules. The MD description of regime I shows that water can cross the membrane in limited amount due to a very restricted passage but it is not enough to impact the cell volume. In the presence of 20 vol% of DMSO (5.97 mol%), the plasma membranes were affected enough to be permeabilized and allow the entry of water as the cells exhibited a round and swollen shape with smooth membranes. The stable pores of regime II allow for an unrestricting crossing of the membrane and a net influx of water that is observed in the experiments with cells.

In the presence of 30 vol% of DMSO (9.79 mol%), numerous blebs were observed on the plasma membranes suggesting a dissociation between the membrane and the cytoskeleton and thus indicating a stronger effect of the DMSO on the membrane structure than for lower concentrations of DMSO. Plasma membrane blebs are dynamic cell protrusions that have been implicated in several biological phenomena (apoptosis, cytokinesis, cell movement, electropermeabilization, …) [25], [26]. Considering the generation of these blebs, the associated global cell volume increase also reflects DMSO-induced membrane permeabilization.

The three regimes found in the MD simulation are thus characterized in these experiments. At low concentrations of DMSO (e.g. 10 vol%), the undulations of the plasma membrane are in agreement with the decrease of membrane thickness and the concomitant increase of membrane area described in the regime I of the MD simulations. At an intermediate concentration of DMSO (e.g. 20 vol%), the permeabilization to water (revealed by the round shape of the cells and their volume increase) is in agreement with the apparition of stable pores as explained in the regime II. At higher concentrations of DMSO (more than 30 vol%) the observed blebs reveal cell membranes damages in accordance with the regime III of the MD simulations.

**Figure 8:**
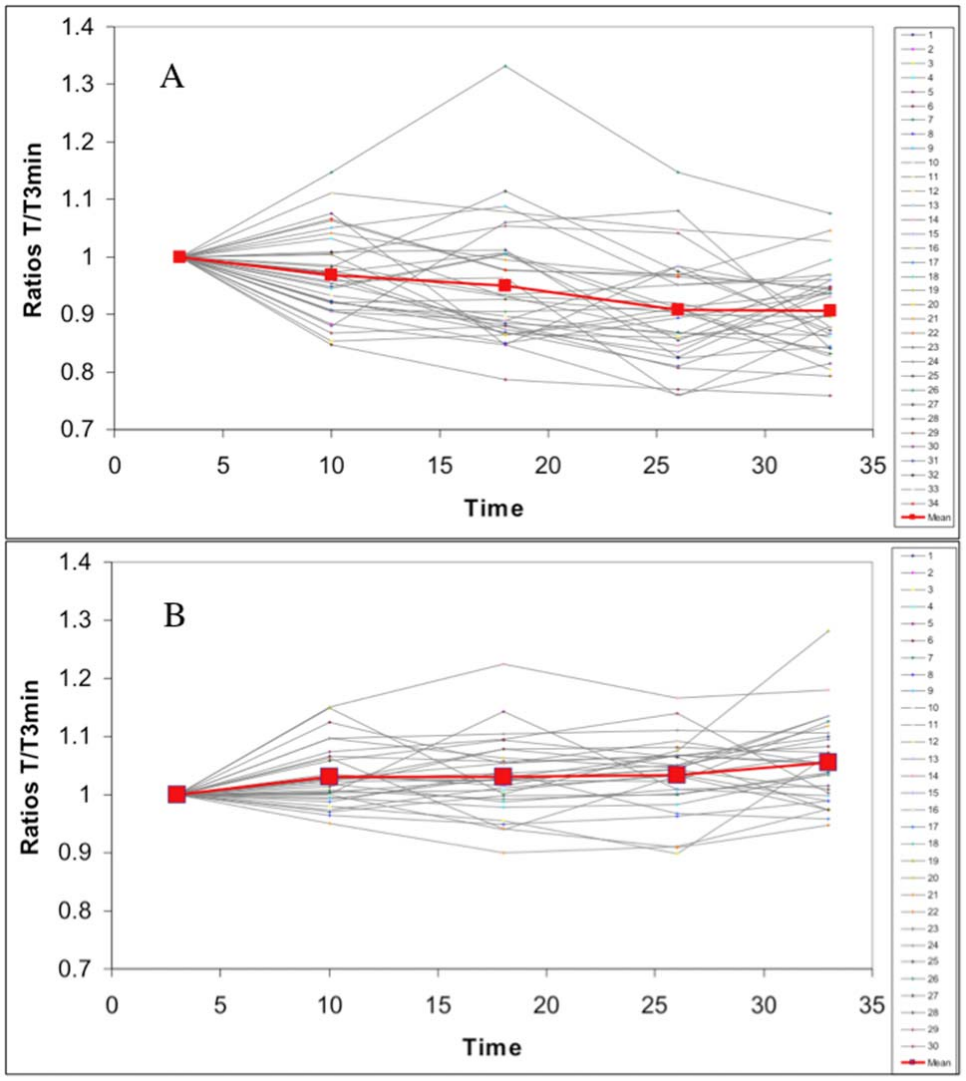
Evolution of diameters of DC-3F cells. Values taken during 30 min (from 3 to 33 min) in the presence of 10 vol% (A) and 20 vol% (B) of DMSO. The cells are incubated with complete MEM and DMSO.

A quantitative approach of the water permeabilization has been done by following the relative cell volume changes throughout the incubation in the presence of DMSO (Figure 8). Actually, the diameter of cells in suspension was measured 3 and 33 minutes after the addition of the DMSO. It was not possible to measure the cell diameter at earlier times after the addition of the DMSO because the cells, which are round and not attached to their support, had to stabilize and stop moving before start taking the picture on which diameters were later measured. Pictures were taken every minute during 30 minutes and were used to quantitatively investigate the relative volume changes of the cells. At 10 vol% DMSO, this quantitative approach revealed that cells diameters shrink by 9.4% (p<0.001). The shrinking of the cells is the consequence of an hyperosmotic shock since the addition of DMSO causes an increase in the osmolarity of the solutions [27]. At 20 vol% DMSO, cells diameters enlarged by 5.6% (p<0.001). This cell swelling suggests that there is a membrane permeabilization sufficient to allow the entry of the DMSO (MW = 78 g/mol) as proposed by the MD simulations. Under these conditions, exchanges of small ions should also occur resulting in an unbalanced osmotic pressure due to the so-called colloid osmotic mechanism [28], [29]. Shortly, small soluble particles can freely cross the porated membrane to balance the electrochemical, osmotic and concentration gradients. Conjointly, larger soluble particles that cannot cross the membrane are trapped inside the cell and unbalance the osmotic pressures leading to a water entry. Actually, penetration of Ca^2+^ ions will be shown in the next section. Experiments could not be reasonably conducted at 30 mol% because the cell morphology was too much altered (presence of blebs that prevents accurate measurements of the cell diameters).

#### Permeabilization to calcium at different concentrations of DMSO

We used the Fluo-4-AM calcium marker to monitor the cytosolic calcium concentration and evidence the cell plasma membrane permeabilization to Ca^2+^. The cells were loaded with the calcium marker Fluo-4-AM which possesses acetoxymethyl groups (AM) allowing the molecule to enter the cell and preventing calcium binding outside of the cell. Once the marker is inside the cytoplasm, the AM groups are cleaved by endogenous esterases resulting in both the trapping of the fluorophore and its activation.

**Figure 9:**
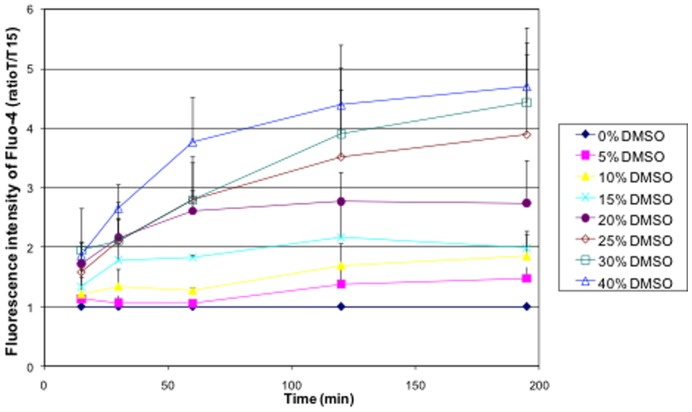
Kinetics of the Ca^2+^ uptake by DC-3F cells in the presence of different concentrations of DMSO (From 0 vol% to 40 vol%). DMSO was diluted in complete MEM. Data collected by flow cytometry. Error bars correspond to standard deviations calculated from three independent experiments.

The kinetics of the Ca^2+^ permeabilization induced by the DMSO is reported on Figure 9, the increase in Fluo-4 fluorescence is revealing the increase in intracellular Ca^2+^ concentration (this calcium is coming from the external medium MEM which contains 1.8 mM of Ca^2+^). The values are ratios between the fluorescence intensity of the sample and the fluorescence of the control measured at the same time to account for the photobleaching of the marker throughout the experiment. The kinetics was dose-dependent and the maximum value of Fluo-4 fluorescence depended on the DMSO concentration. For DMSO concentrations of 15 vol% and above, Ca^2+^ accumulation seems increasingly faster with increasing DMSO concentrations. Actually, taking the values at 15 minutes and 30 minutes, the extrapolation to the base line (ratio 1) occurs at time zero for most of the DMSO concentrations (10 vol%, 15 vol%, 25 vol% and 40 vol%). This means that calcium starts entering the cells immediately after the beginning of the exposure to the DMSO. It can also be speculated that the larger the DMSO concentration, the larger the membrane perturbation and the faster the Ca^2+^ uptake as shown in the Figure 9. After 60 minutes of exposure to the DMSO, there is a slow down in the Fluo-4 fluorescence increase and even a plateau of the fluorescence value in the cases of 15 vol% and 20 vol% of DMSO. This plateau could be due to the fact that a stationary state in the cytoplasmic Ca^2+^ concentration is generated between the calcium entering the cell through the DMSO-affected membrane and the normal storage of the calcium in the endoplasmic reticulum reservoirs. Indeed, intracellular Ca^2+^ concentration is a cellular parameter strongly regulated in cells. Therefore, these results suggest that the DMSO allows the increase of the intracellular Ca^2+^ concentration by making the membrane permeable to the Ca^2+^ ions and this difference in Ca^2+^ concentration induces the activation of the Ca^2+^ intracellular concentration regulation mechanisms.

Because the flow of Ca^2+^ at 20 vol% of DMSO should be larger than the flow at 15 vol% of DMSO, resulting in a higher concentration at the stationary state it can be deduced that Fluo-4 (MW = 736 g/mol) does not leak the cells through the DMSO-permeabilized membrane at least during the first 180 minutes after the addition of the DMSO. However, it was not possible to exclude an interaction of the DMSO with the Fluo-4 that could be responsible for the difference in the fluorescence plateau values as a function of the DMSO concentration. The influence of DMSO on the Fluo-4 fluorescence what thus determined. The DMSO increases this fluorescence (Data S1) but the most important enhancement of the signal due to this phenomenon at 40 vol% of DMSO is less than a 1.5-fold increase in comparison to the 0 vol% of DMSO. In Data S1 we show that for 40 vol% of DMSO, after 180 min, there is a 4.7 fold increase in the fluorescence which cannot be explained just by the action of the DMSO on the Fluo-4.

In conclusion, the three regimes were also detectable in the Ca^2+^ uptake experiments. The regime I, corresponding to less than 10 vol% of DMSO, where there is no stable pore formed, results in almost no increase in fluorescence, at least at short incubation times. The regime II (15 vol% and 20 vol% of DMSO) displays stable pores and results in membrane permeabilization to Ca^2+^. The lack of plateau in the presence of 25 vol%, 30 vol% and 40 vol% of DMSO seems to show a perturbation too strong to be counter-balanced by the cell. This is consistent with the regime III and the description of a destruction of the membrane configuration.

#### Permeabilization to Yo-Pro-1 at different concentrations of DMSO

**Figure 10:**
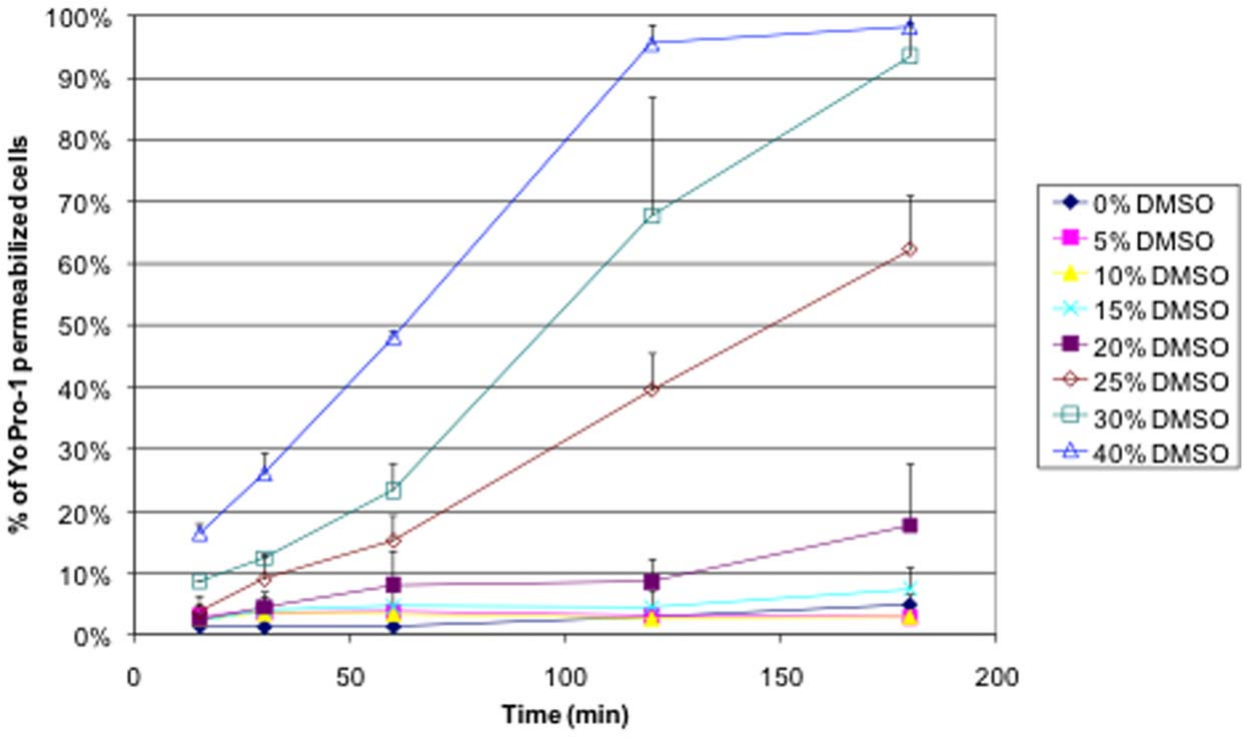
Kinetics of the Yo-Pro-1 uptake by DC-3F cells in the presence of different concentrations of DMSO (From 0 vol% to 40 vol%). DMSO was diluted in complete MEM. Data collected by flow cytometry. Error bars correspond to standard deviations calculated from three independent experiments.

Yo-Pro-1 is unable to cross the intact membranes. If it may enter the cell, as a DNA-intercalant dye it binds to DNA and become much more fluorescent than the free Yo-Pro-1 [23], [24]. The permeabilization of cells by the DMSO is clearly detectable using the Yo-Pro-1 (Figure 10). First of all, there is no measurable permeabilization up to 15 vol% of DMSO after 3 hours of incubation in the presence of DMSO. At 15 vol% and 20 vol% of DMSO, no or very small amounts of Yo-Pro-1 enter the cells. Finally, above 20 vol% of DMSO, the higher the concentration of the DMSO, the faster and the more extended the cell permeabilization. Again, Yo-Pro-1 uptake seems to start immediately after the addition of DMSO.

Because Yo-Pro-1 strongly binds to DNA when it enters the cells it cannot go out anymore and the Yo-Pro-1 fluorescence cannot further decrease as no mechanism can exclude the Yo-Pro-1 after its binding to the DNA. This is why all the conditions in which DMSO permeabilizes the cell membrane are tending to the same percentage of Yo-Pro-1 permeabilized cells (100%) whereas, in the Figure 9, the maximum value of Ca^2+^ concentration of each sample depends on the DMSO concentration.

The same regimes as the ones described in the Ca^2+^ experiments and the MD simulations are found here. There is no detectable permeabilization up to 10 vol% of DMSO (regime I). At 15 vol% and 20 vol% of DMSO a reduced permeabilization is measured, in agreement with the fact that Yo-Pro-1 is a molecule larger than the Ca^2+^ (regime II). In the presence of 25 vol%, 30 vol% and 40 vol% of DMSO, (regime III), the permeabilization is fast and starts after the contact with the DMSO.

### Conclusions

In this paper we have presented a combination of numerical and experimental results regarding the effect of DMSO on cell permeability, a phenomenon with important consequences in many procedures in biotechnology and medicine that require the transfer of chemicals inside the cells through their plasmatic membrane.

It is important to notice, that our MD simulations have been performed on phospholipids bilayers containing Chol. In animal cells, there is always Chol in the membrane lipid mixture, and since this compound is fundamental for determining many structural properties of the cell membrane, and in particular its permeability to small molecules, Chol-containing membrane is a better model to study permeabilization effects than single-lipid bilayer. Summarizing, our simulations reveal that DMSO counteracts the condensing effect of Chol in the membrane properties: whereas Chol promotes membrane packing, ordering and cohesion, DMSO has the opposite effect. Additionally, the three modes of action of DMSO (membrane loosening, pore formation and bilayer collapse) reported in previous MD studies for single-lipid bilayers [7]–[11] are captured here for Chol-containing systems. This finding constitutes a clear progress in order to validate the applicability of the numerical observations regarding the effect of DMSO to biological cell membranes.

The most relevant effect of DMSO is its ability to form hydrophilic (stable) pores in a lipid bilayer. Detailed inspection of the pore formation process has revealed that DMSO provides an exclusive mechanism of membrane permeability whose molecular details have been unveiled in this paper. Due to its small size and amphiphilic nature, DMSO molecules are located in the inner membrane/water interface region, stabilizing the initial water fluctuations and subsequent hydrophobic water columns, protecting them from the hydrophobic membrane environment. The general characteristics of this mechanism based on a specific DMSO surfactant-like action are rather independent of the nature of the lipid bilayer. It has been reported here for our DOPC/20%Chol bilayers, but it has been also captured in membranes containing up to a 40 mol% (not shown).

Experiments with living cells have qualitatively confirmed the results from numerical simulations and have demonstrated the feasibility of DMSO-induced membrane permeabilization in living cells. At low DMSO fractions (<15 vol%), membranes display undulations that may correspond to the regime I described in the simulations. No water, Ca^2+^ or Yo-Pro-1 entrance is significantly accounted in this regime. At intermediate DMSO fractions (15 vol% and 20 vol%, in the experiments reported here) water and Ca^2+^ enter the cell as detected by the cells swelling and by the Fluo-4 fluorescence increase respectively. This situation may correspond to the regime II characterized in the MD simulations with the generation of stable pores in the membrane. At higher DMSO fractions (>20 vol%) cells are presenting numerous extended blebs suggesting a loss in the membrane integrity leading to membrane destruction as shown in the regime III. In this case, cell plasma membrane permeabilization is easily detected even with the Yo-Pro-1 molecule. It is noticeable that the concentrations of DMSO used in cryopreservation, between 5 vol% [30] to 20 vol% of DMSO [31], are corresponding to regimes I and II which preserve cell integrity. It can be easily understood that larger DMSO fractions cannot be used because of their toxicity.

To conclude, we have presented a comparison between atomistic simulations and experiments with real cells that validates for the first time the DMSO regimes of action in Chol-containing membranes and the qualitative verification of these regimes in real cells. Notice that simulated membranes are extremely simplified membrane systems, far for the complexity of biological plasma cell membranes (inserted proteins, lateral organization in rafts, attachment to cytoskeleton, etc.), and as a consequence any attempt to make quantitative predictions is not achievable. However, real qualitative behaviors and trends can be captured with numerical simulations as shown in this paper.

## Materials and Methods

### Molecular Dynamics Simulations and Protocols

The MD simulations were performed using the GROMACS v.3.3.1 software package [32]. For DOPC molecules we used the standard united-atom force-field parameters developed by Berger et al. [33]. The partial charges were taken from Ref. [34] and the force field parameters for the double-bond region are based on the adaptation performed by Bachar et al. [35], [36]. The Simple Point Charge (SPC) model was employed for water. The force field parameters of Holtje et al. [37] were used for Chol. The DMSO force field description developed by Bordat et al. [38] was used here as in a number of other recent papers reporting MD simulations with DMSO [9], [10]. The SETTLE algorithm was used to preserve the bond lengths in water molecules, whereas the lipid and DMSO bond lengths were constrained with the LINCS algorithm. A single 1.0 nm cut-off distance was used for the Lennard-Jones interactions. The long-range electrostatic interactions were handled using the particle-mesh Ewald method with a real space cut-off of 1.0 nm, **β**-spline interpolation (of order 6), and direct sum tolerance of 10^−5^. Periodic boundary conditions were used in all three directions, and the time step was set to 2 fs.

The simulations were carried out in the NpT ensemble at p = 1 atm and T = 310 K. In these conditions, all simulated membranes displayed a fluid state. The temperature and pressure were controlled by using the weak coupling method [39] with the relaxation times set to 0.6 and 1.0 ps, respectively. The pressure coupling was applied separately in the bilayer plane (*xy*) and the perpendicular direction (z).

### Description of the Simulated Systems

We carried out atomic-scale MD simulations for DOPC membrane systems mixed with 20 mol% of Chol. All bilayers were composed of 128 DOPC molecules together with 32 Chol molecules, homogeneously distributed in the two leaflets. All DMSO-free systems were sufficiently hydrated with 6186 water molecules, and their initial configurations were taken from previously equilibrated DOPC bilayers from Ref. [40].

Bilayers with different amounts of DMSO were also simulated. In all cases, the number of solvent molecules was fixed to 6186, but the molar fraction of DMSO in the water/DMSO mixture was varied. Each bilayer system was run for 1, 2.5, 5, 7.5, 10, 15, 20, 40 and 60 mol% of DMSO were run for DOPC/20%Chol membranes. DMSO molecules were included in the equilibrated pure lipid membranes in three different manners: randomly in the whole system or as a DMSO box in the water/membrane interface or in the aqueous bulk phase. In the three cases, DMSO molecules were partially absorbed by the bilayer and the equilibrium is taken when DMSO density profiles were independent of the initial insertion place and remained constant after a few nanoseconds.

Equilibration of the bilayers was determined by monitoring the membrane area and the DMSO density profile. The simulated membranes are equilibrated after 20 ns, and all temporal averages for the computation of membrane properties were performed in a period of 25 ns after equilibration was reached. The simulation protocol applied here has been successfully applied in previous MD simulations [36], [40], and the obtained values for structural membrane properties such as the area per molecule, the membrane thickness, and the scattering form factors have been well in line with experimental data for pure DOPC bilayers and their counterparts with 20 mol% of Chol [41].

### Cell Line and Culture Conditions

DC-3F cells (Chinese hamster lung fibroblast cells) [42] were grown in MEM (Minimum Essential Medium) supplemented with 10% fetal bovine serum, 100 U/mL penicillin and 100 µg/mL streptomycin. The cells culture chemicals were purchased from Invitrogen (Cergy Pontoise, France) and the DMSO from Sigma-Aldrich (St Quentin Fallavier, France). Cells were propagated at 37°C in a humidified 5% carbon dioxide atmosphere.

### Cell Imaging and Size Measurements

For the observation of the DC-3F cells morphology in the presence of different concentrations of DMSO, a confocal microscope Leica TCS SPE with an objective ACS APO 63x, 1.30 NA oil and the LAS AF software version 2.4 (Leica, Germany) was used.

For the cell size measurements, DC-3F cell images were taken with a Zeiss AxioCam Hrc and Axio Vision 4.6 software (Carl Zeiss, Germany) on a Zeiss Axiovert S100 epifluorescence inverted microscope. The diameters (D_T_) of the same cells were measured after 3 (D3 min), 10, 18, 26 and 33 minutes of incubation in DMSO. The ratio D_T_/D3 min is reported.

### Flow Cytometry and Calcium Permeabilization

Adherent DC-3F cells were incubated 30 minutes in the presence of 5 µM of Fluo-4 AM (λex  = 496 nm, λem  = 515 nm, Invitrogen) at 37°C and in a humidified 5% carbon dioxide atmosphere. Then the cells were detached from the culture support using Tryple Express solution (Invitrogen), centrifugated and suspended in MEM containing DMSO from 0% to 40% vol/vol (DMSO to water molar ratio ranging from 0% to 14.5% mol/mol). Cells were incubated under constant agitation at 37°C and in a humidified 5% carbon dioxide atmosphere. 10^4^ cells were analyzed in a C6 Flow Cytometer Accuri (USA) at different times.

### Flow Cytometry and Yo-Pro-1 Permeabilization

Adherent DC-3F cells were detached from the culture support using Tryple Express solution, centrifugated and suspended in MEM in the presence of 5 µM of Yo-Pro-1 (λex  = 491 nm, λem  = 509 nm, Invitrogen), used as an indicator of membrane permeabilization, and DMSO from 0% to 40% v/v (DMSO to water molar ratio ranging from 0% to 14.5% mol/mol). Cells were incubated under constant agitation at 37°C and in a humidified 5% carbon dioxide atmosphere. 10^4^ cells were analyzed in a C6 Flow Cytometer Accuri at different times.

All the experiments have been done 3 times and the results are expressed as means ±SD.

## Supporting Information

Data S1 **Influence of the DMSO on the Fluo-4 fluorescence.**
(DOC)
