# Supplementary material for: Effects of Dimethyl Sulfoxide in Cholesterol-Containing Lipid Membranes: A Comparative Study of Experiments In Silico and with Cells
Source: PLoS One. 2012 Jul 25;7(7):e41733. doi: 10.1371/journal.pone.0041733 (PMC3404987; doi:10.1371/journal.pone.0041733)
Supplement: Data S1 — Influence of the DMSO on the Fluo-4 fluorescence. (DOC) [file pone.0041733.s001.doc]

Supplementary data

| 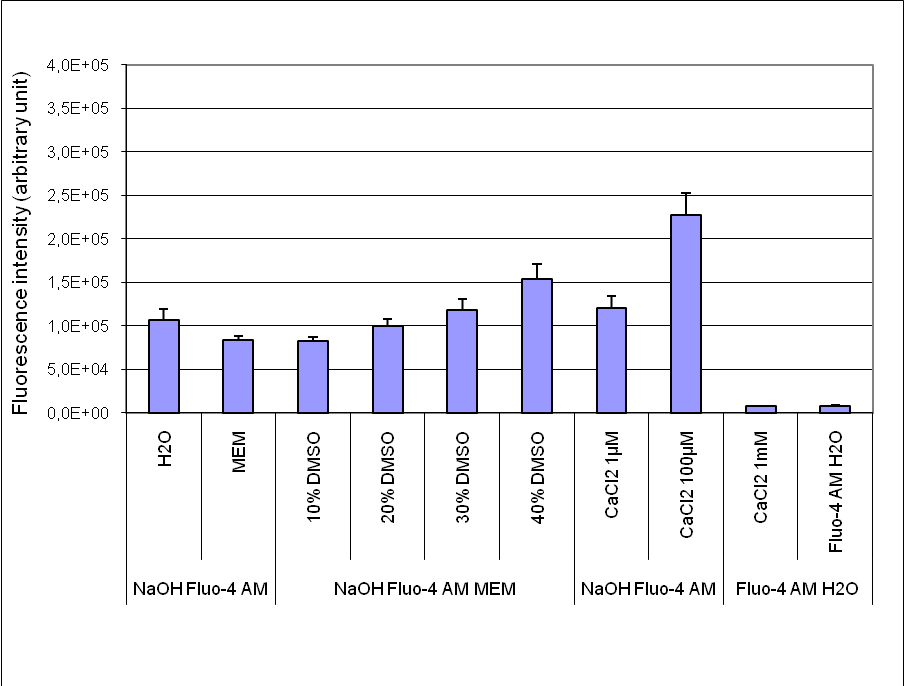 |
| --- |

**Figure S1. Influence of the DMSO on the Fluo-4 fluorescence.** The Fluo-4 AM needs to be cleaved and activated to react with the Ca^2+^ ions. In cells, esterases are cleaving the AM groups but in this experiment NaOH has been used to activate the dye. The Fluo-4 fluorescence assays have been done in 96 wells plates and read by a fluorescence plate reader (Glomax multi+ detection system, Promega, France). The CaCl_2_ has been used as positive control for the Fluo-4 activation. The use of water instead of NaOH is a negative control because in this case the AM moiety is not cleaved and therefore Fluo-4 AM is not activated.

An initial solution of NaOH (90 µM) and Fluo-4 AM (10 µM) is prepared. 50µL of this solution were distributed in the wells of a 96 wells plate. After one hour of incubation at room temperature, in the dark, 50µL of complete MEM (10% FBS, 1% penicillin and streptomycin) with 20% to 80% v/v of DMSO was added in wells in order to get 10%, 20%, 30% and 40% of DMSO for the final concentration. After the addition of water, complete MEM (with or without DMSO) or CaCl_2_, all the wells contained 100 µL of solution.

The DMSO concentration influences Fluo-4 fluorescence with a 1.5-fold increase at the maximum amount of DMSO (40%).
